# Cattell-Braasch maneuver combined with local hypothermia during superior mesenteric artery resection in pancreatectomy

**DOI:** 10.1007/s00423-016-1501-5

**Published:** 2016-08-25

**Authors:** Sofia Westermark, Elena Rangelova, Christoph Ansorge, Lars Lundell, Ralf Segersvärd, Marco Del Chiaro

**Affiliations:** Pancreatic Surgery Unit, Division of Surgery, Department of Clinical Science, Intervention and Technology (CLINTEC) Karolinska Institutet at Center for Digestive Diseases, Karolinska University Hospital, K53, 14186 Stockholm, Sweden

**Keywords:** Resection of superior mesenteric artery, Pancreatectomy associated to vascular resection, Pancreas cancer, Pancreas surgery, Vascular resection, Artery resection

## Abstract

**Background:**

The recent development of new neo-adjuvant treatment regimens associated with a higher success rate of down-staging has increased the interest of pancreatic surgeons on the role of extended surgery for patients affected by locally advanced pancreatic cancer. Pancreatectomy together with resection of the portal/superior mesenteric vein is considered nowadays standard of care for patients affected by pancreatic cancer. However, the resection of major abdominal arteries is still debatable. In particular, the short- and long-term results after resection of the superior mesenteric artery (SMA) remain controversial and only few cases have been described in literature. The present paper describes a new, quick, and easy technique for resection of the SMA.

**Clinical case:**

A 71-year-old patient affected by IPMN cancer with infiltration of the SMA received FOLFIRINOX-based neo-adjuvant treatment. After 4 months of treatment, the patient underwent total pancreatectomy with en bloc resection of the SMA and direct end-to-end anastomosis. The vascular resection was performed combining a complete Cattell-Braasch maneuver with local bowel hypothermia in an attempt to avoid graft interposition by facilitating an end-to-end anastomosis and to reduce the warm ischemia time. The post-operative course was uneventful and the patient was discharged 8 days post-operatively.

## Background

At the time of diagnosis, about 30 % of the patients with pancreatic ductal adenocarcinoma (PDAC) are excluded from surgical resection due to locally advanced disease [1]. Recently, the International Study Group for Pancreatic Surgery published a consensus paper in which the resection of the superior mesenteric/portal vein (SMPV) is considered standard of care in case of limited tumor encasement [2]. There is much less evidence, however, regarding the value of the arterial resections in PDAC. In a meta-analysis, Mollberg and co-workers stated that pancreatectomies with arterial resections are associated with a poor short- and long-term outcome [3]. Recent data from literature, however, implies that the short-term outcome of arterial resections performed in expert hands is comparable to that of conventional pancreatectomies [4]. Other data shows that long-term results are better for isolated arterial resections only rather than combined arterial-venous ones [1]. The advantageous long-term outcome probably reflects the fact that a single-artery contact is an expression of a tumor occurring in an unfavorable location rather than a locally advanced tumor growth, involving multiple vessels. Furthermore, published data shows that, for patients with the same stage of disease, the long-term prognosis is better if they are treated with pancreatectomy with isolated arterial resection compared to palliative chemo (-radio) therapy.

The recent development of new neo-adjuvant treatment regimens associated with a higher success rate of down-staging [5] has increased the interest of pancreatic surgeons on the role of extended surgery for patients affected by locally advanced pancreatic cancer. For this reason, the consensus paper from the International Study Group for Pancreas Surgery encourages further investigations of the role of more extensive surgery after neo-adjuvant treatment. The hepatic artery and the celiac trunk are the most frequently resected arteries in pancreatectomy. There are not many cases of superior mesenteric artery (SMA) resections reported in literature [3] and there are multiple reasons for this. One reason is probably that the involvement of the SMA most often reflects a locally advanced tumor growth involving multiple vessels. Also, the resection of the SMA is a technically complex operation associated with several intra- and post-operative life-threatening complications such as bowel ischemia-reperfusion syndrome and SMA thrombosis. The ischemia-reperfusion injury is dependent on two major factors: the duration of ischemia and the temperature of the organ at stake, in this case the intestine [6]. During SMA resection, the duration of ischemia is variable according to different surgical techniques. Generally, the most often used technique is graft interposition from the aorta to the SMA stump in the root of mesentery. This technique generally requires quite a long ischemia time, considering that two anastomoses are needed. Alternatively, a rotation of the splenic artery can be used to rebuild the SMA continuity. End-to-end anastomoses have also been reported, but generally only for resection of a small segment of the SMA [1]. During the clamping time of the SMA, the bowel is exposed to warm ischemia. This case report describes a new original technique for resection of the SMA combining the Cattell-Braasch maneuver and local bowel hypothermia [7], in order to facilitate an end-to-end SMA anastomosis and to reduce the bowel metabolism during the cross clamping of the SMA.

## Case report

**Fig. 1:**
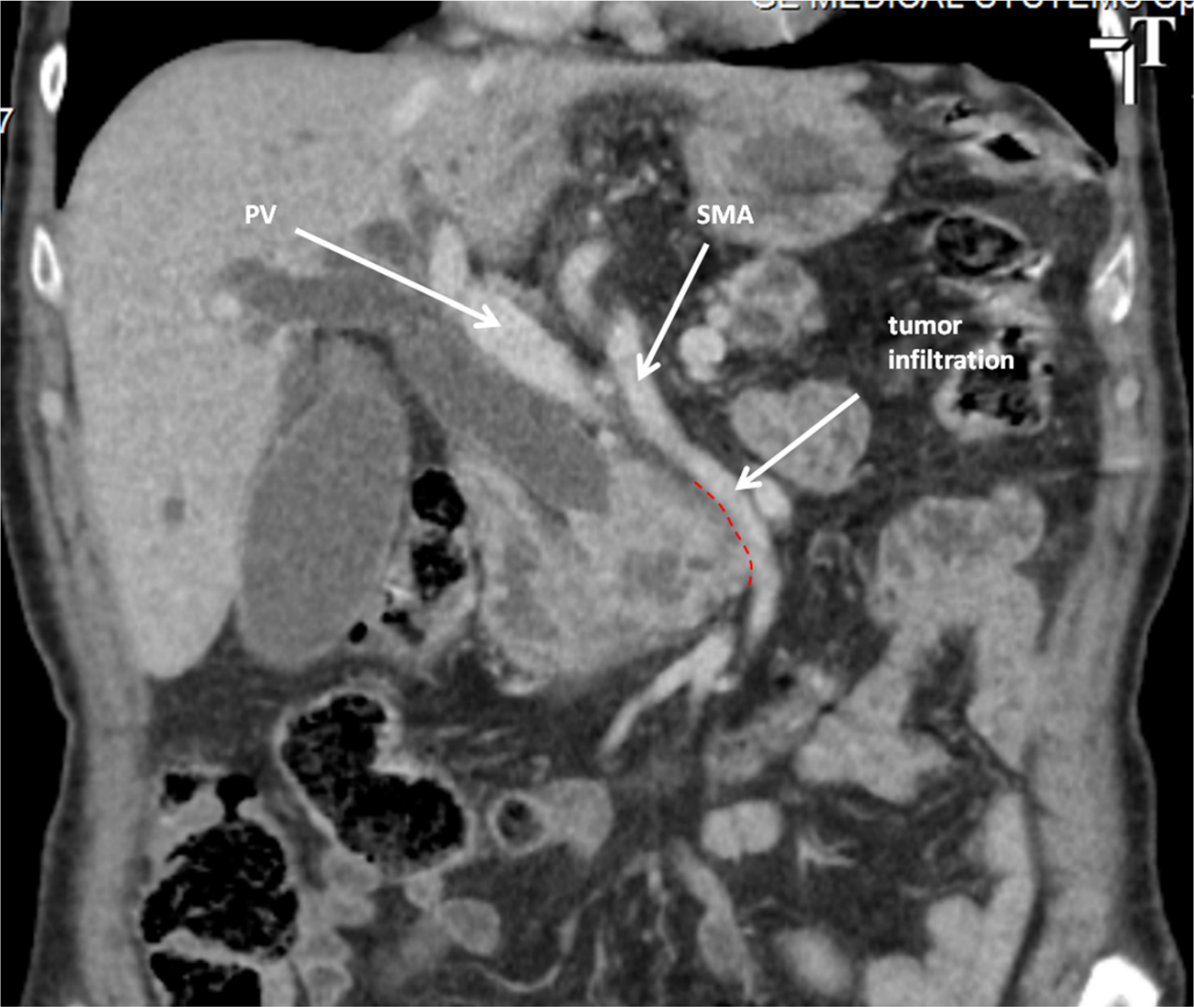
The preoperative CT-scan shows a 3-cm tumor infiltration of the superior mesenteric artery. *SMA* = superior mesenteric artery. *PV* = portal vein

**Fig. 2:**
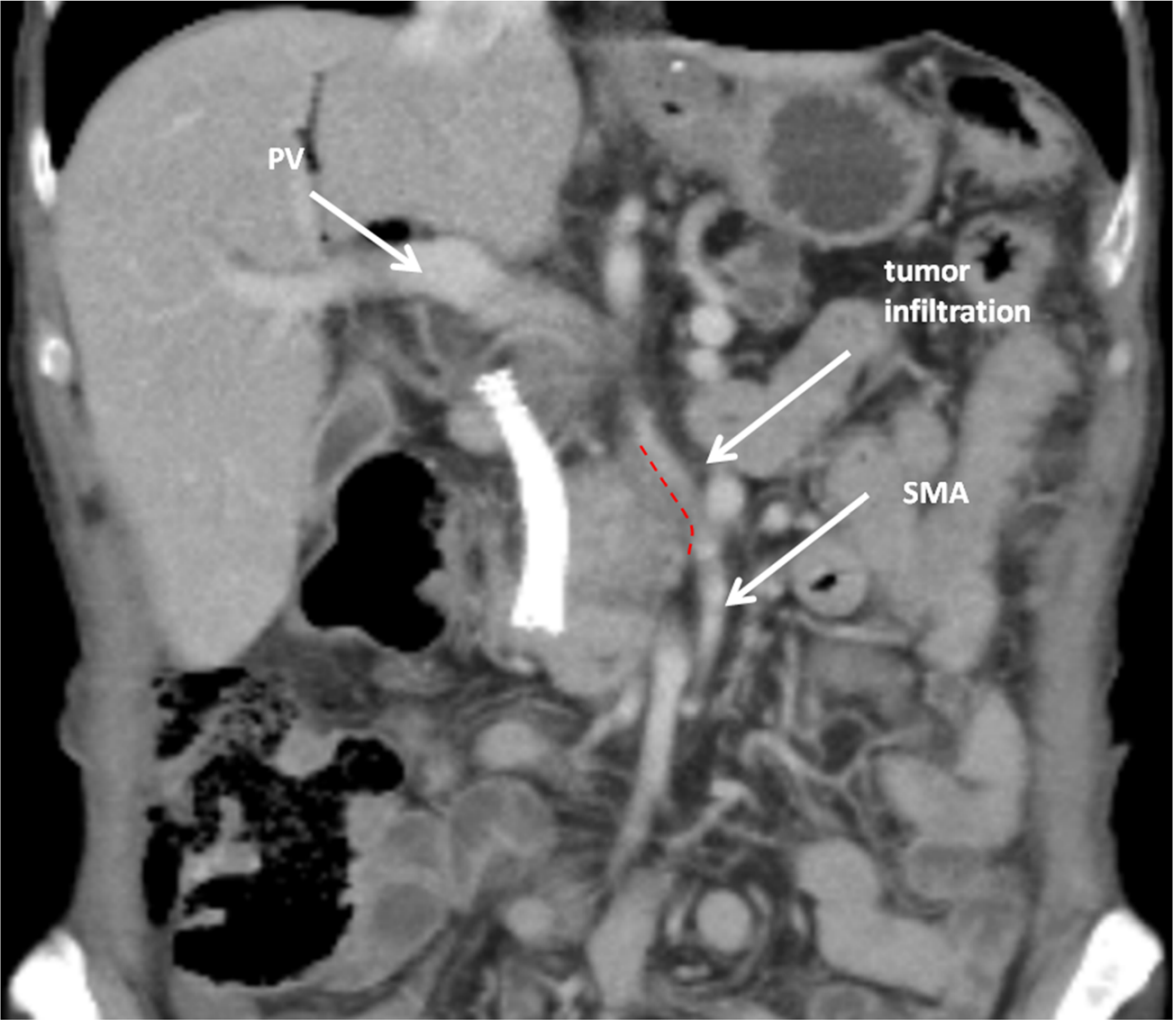
The post neo-adjuvant CT scan shows unchanged tumor encasement of the superior mesenteric. *SMA* = superior mesenteric artery. *PV* = portal vein

**Fig. 3:**
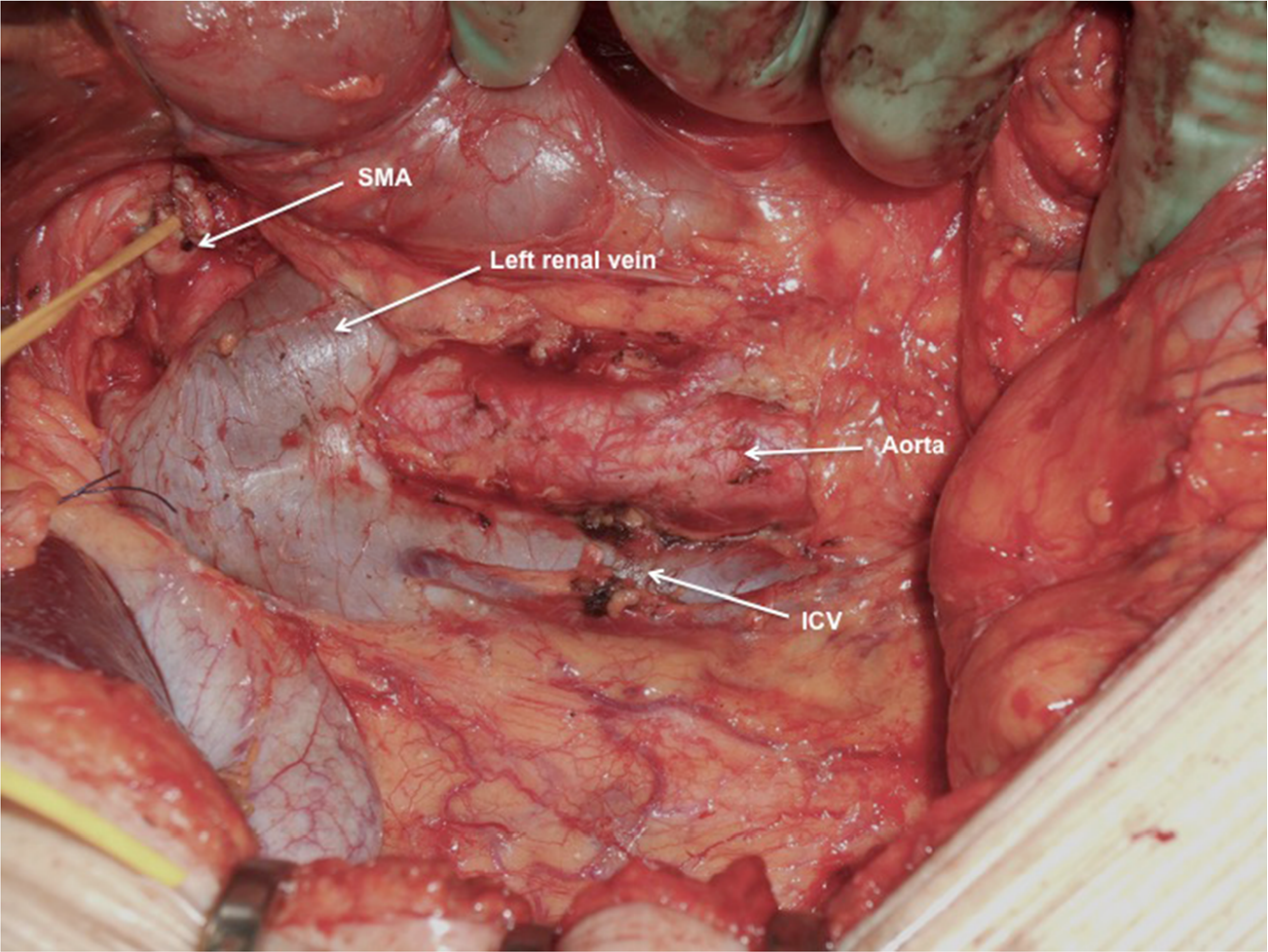
After a Cattell-Braasch maneuver and en bloc aorto-caval lymphadenectomy, the origin of the superior mesenteric artery is identified immediately above the left renal vein. *SMA* = superior mesenteric artery. *IVC* = inferior caval vein

**Fig. 4:**
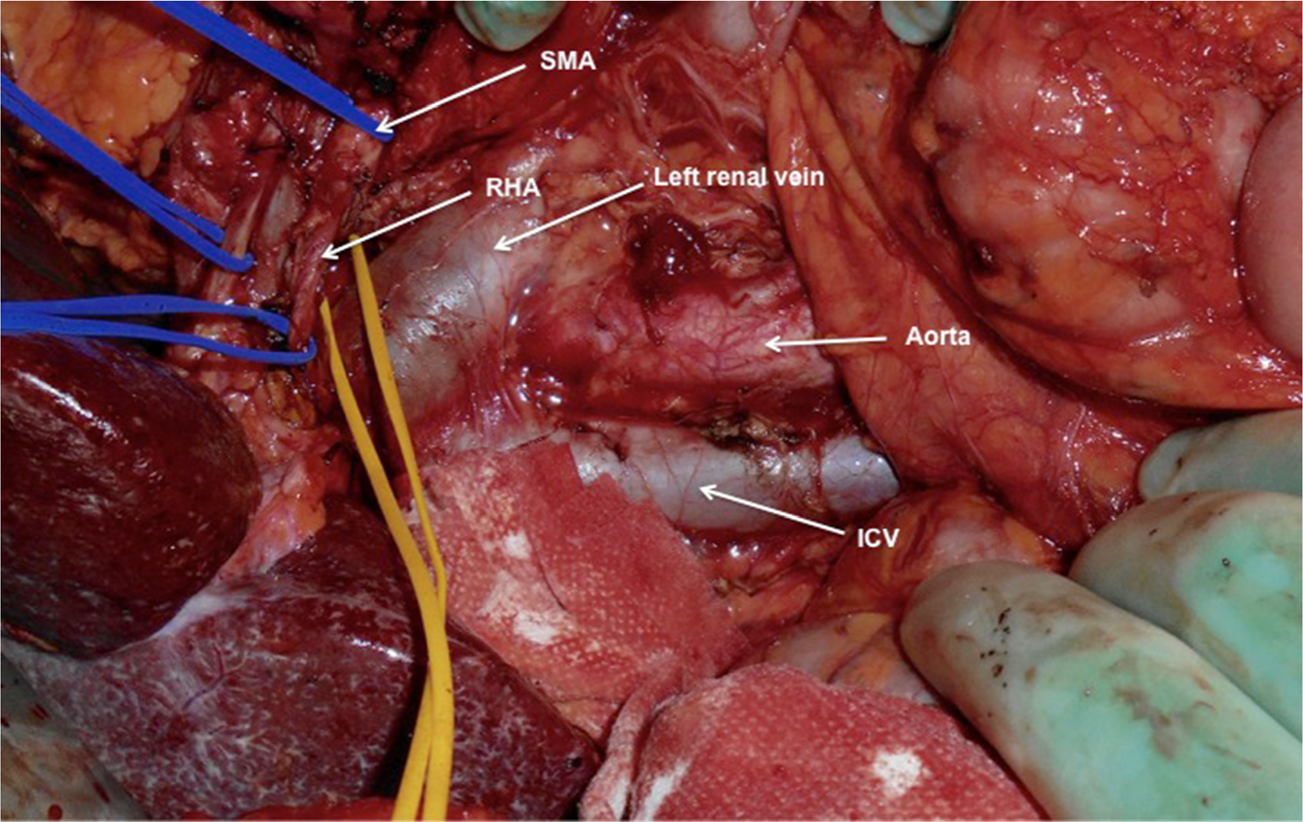
During the anterograde artery-first maneuver, a replaced right hepatic artery originating from the SMA is identified and preserved. *SMA* = superior mesenteric artery. *IVC* = inferior caval vein. *RHA* = right hepatic artery

**Fig. 5:**
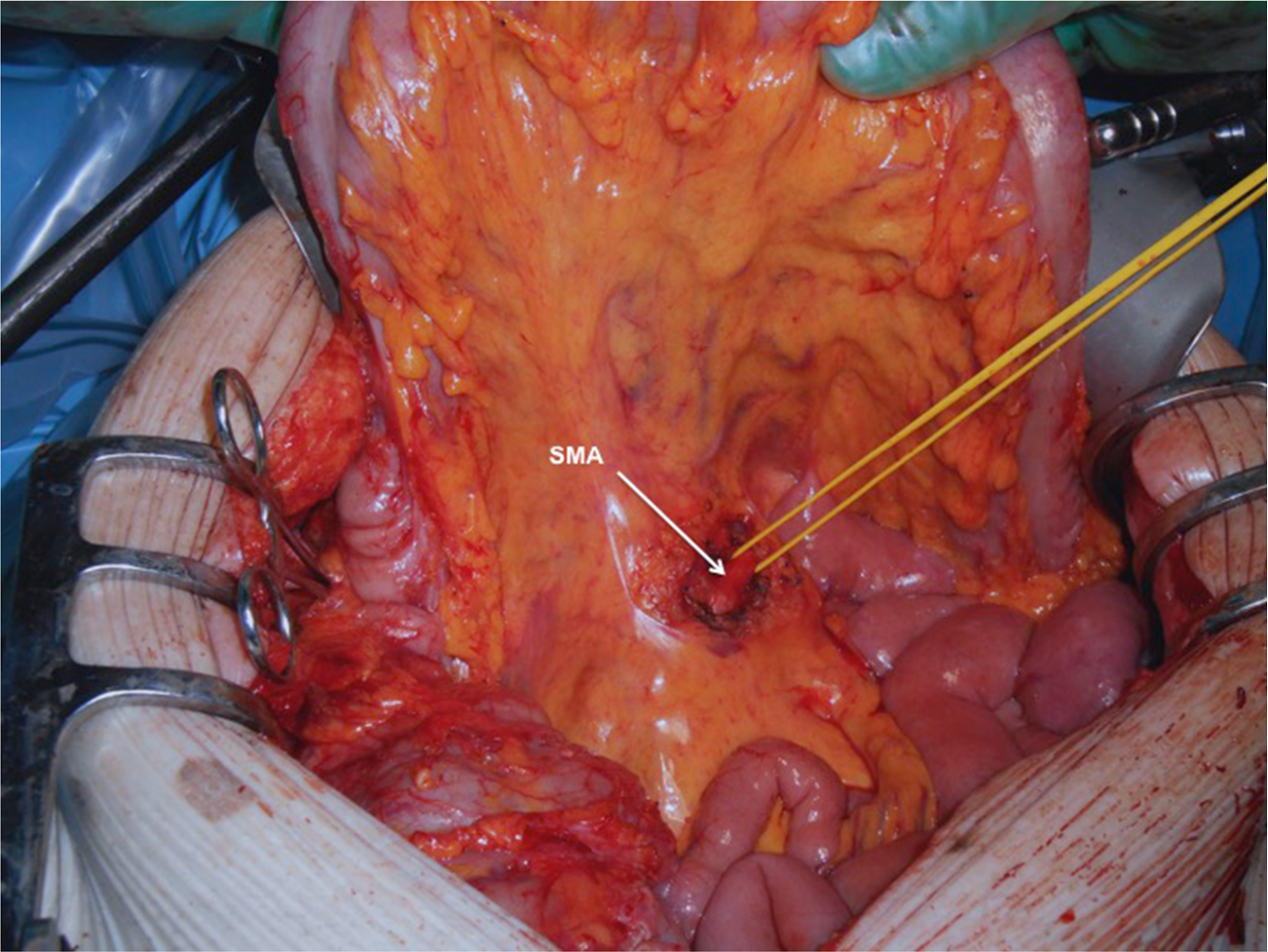
At the point of resectability assessment. The superior mesenteric artery appears free from tumor infiltration in the root of mesentery below the mesocolon. *SMA* = superior mesenteric artery

**Fig. 6:**
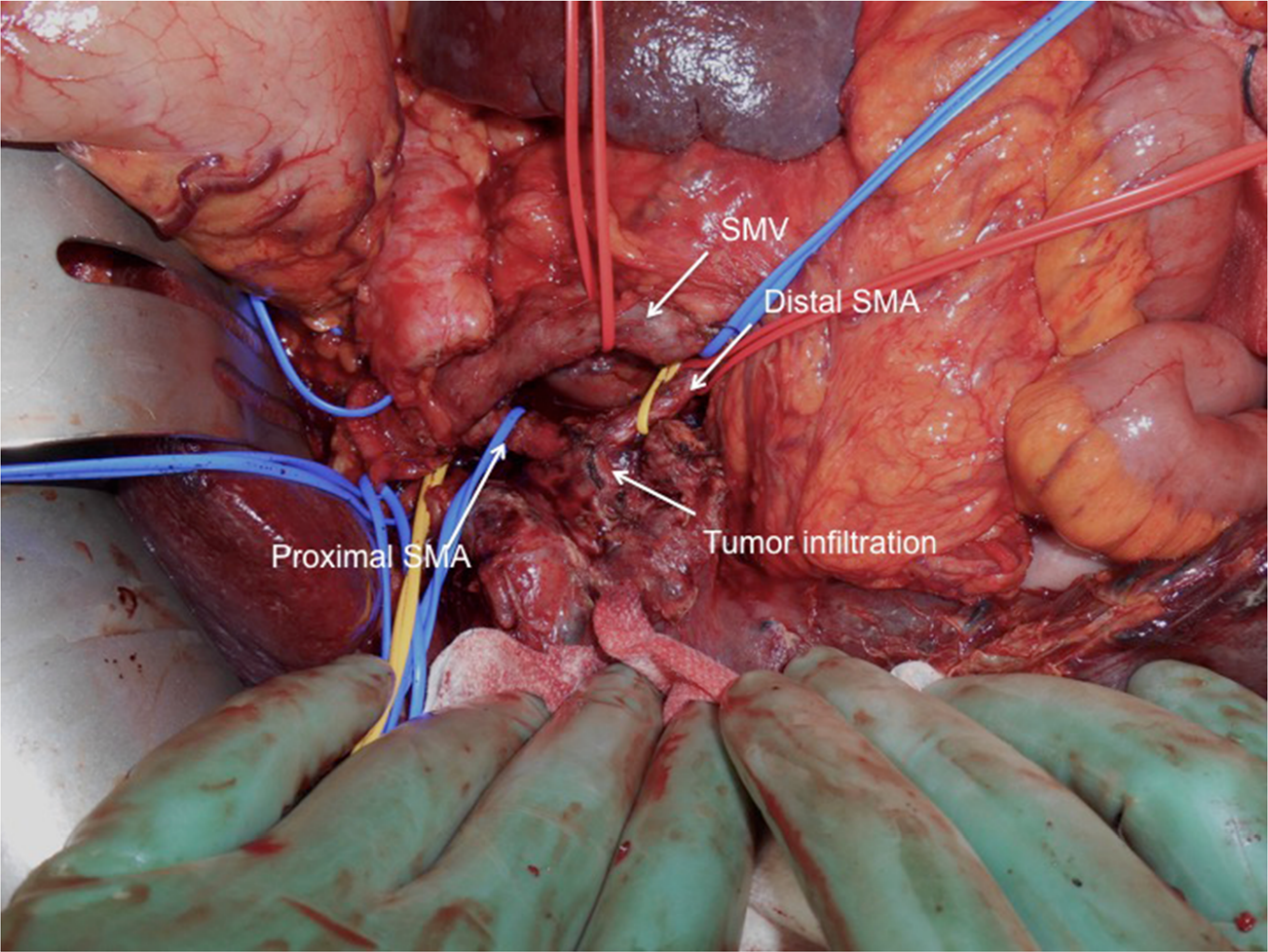
The dissection is completed. The tumor remains attached only on the superior mesenteric artery. The proximal and distal part of the artery is prepared to be clamped to perform an en bloc resection. *SMA* = superior mesenteric artery

**Fig. 7:**
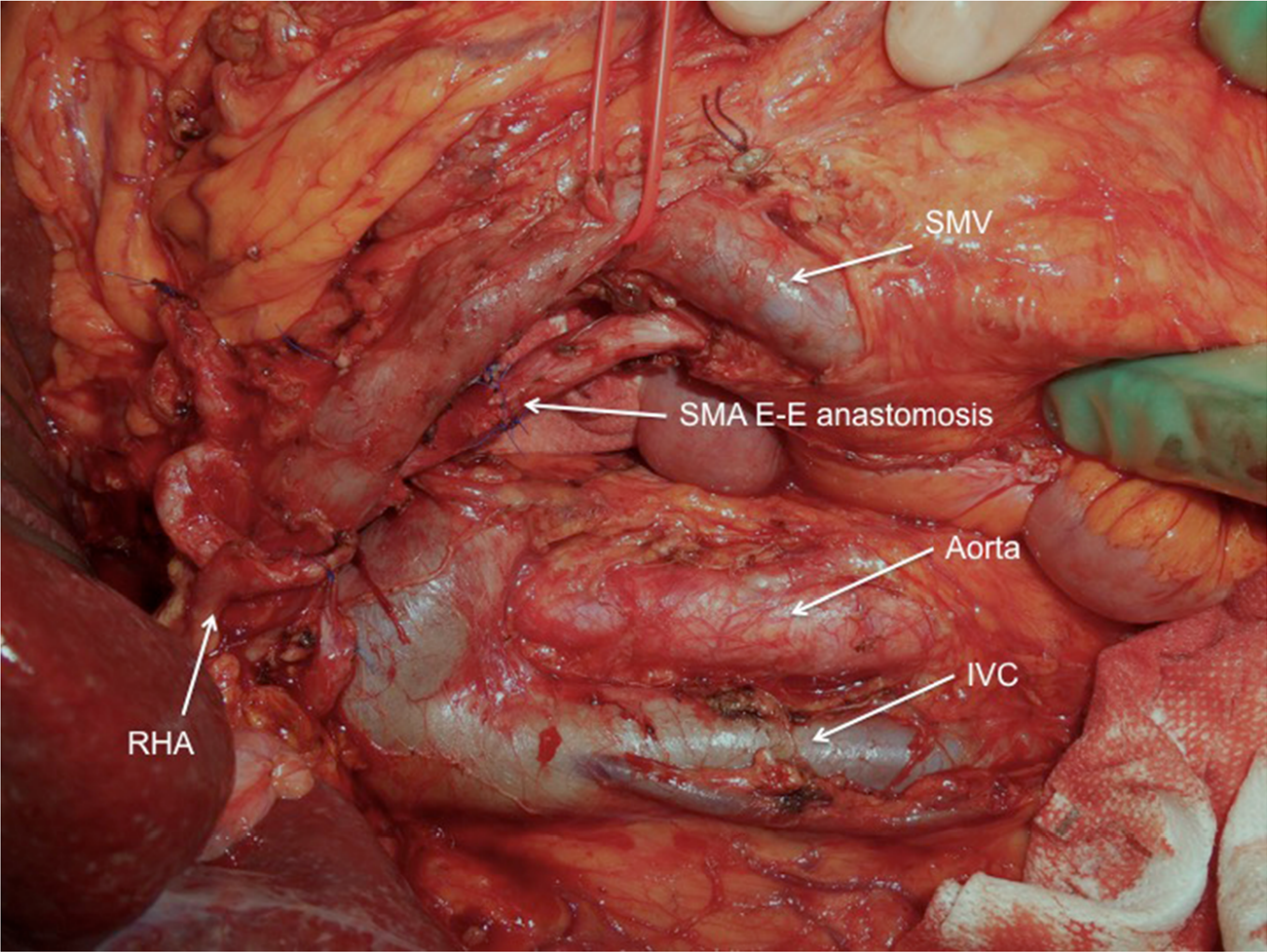
After the en bloc removal of the specimen including a segment of the superior mesenteric artery, the vessel is reconstructed by an end-to-end anastomosis using interrupted Prolene 5/0®. *SMA* = superior mesenteric artery. *IVC* = inferior caval vein. *RHA* = right hepatic artery. *SMV* = superior mesenteric vein

**Fig. 8:**
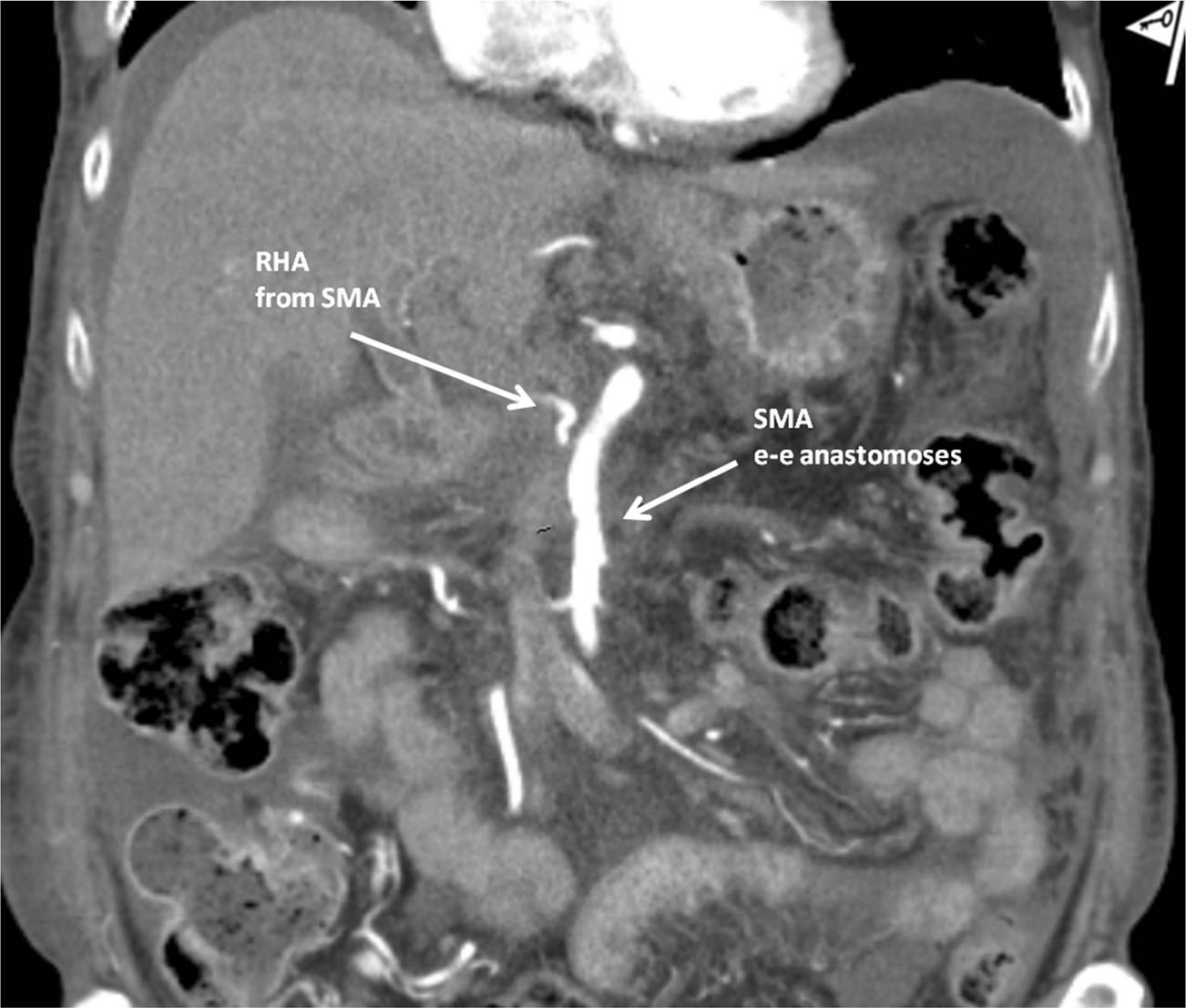
A post-operative CT scan control shows a normal canalization of the SMA anastomosisand flow in the right hepatic artery. *SMA* = superior mesenteric artery. *RHA* = right hepatic artery

A 71-year-old male was admitted with obstructive jaundice at the end of June 2015. A CT scan of the thorax and abdomen demonstrated a mixed-type IPMN with a 50-mm cystic/solid lesion in the head of the pancreas associated with a 3-cm long and more than 50 % circumferential encasement of the SMA (Fig. 1). No distant metastases were detected. The patient was discussed at the Karolinska University Hospital’s multidisciplinary conference. The tumor was considered not primary resectable by oncological means and the patient commenced chemotherapy. He underwent concomitant endoscopic ultrasound-guided tumor biopsy and bile duct stenting. Histology showed IPMN cancer. At the end of July, the patient started neo-adjuvant treatment with FOLFIRINOX [5] that was completed 4 months later without significant toxicity. A new CT scan, demonstrated at the multidisciplinary conference, showed no occurrence of distant metastases and a partial response with a reduced tumor size, however with remaining encasement of the SMA (Fig. 2). As the patient had stable disease, he underwent surgical exploration by a senior pancreatic surgeon (MDC) in December 2015. No distant metastases or ascites were found at the time of surgery. An extended Kocher maneuver was performed with a total mobilization of the right colon and of the small bowel, e.g., Cattell-Braasch maneuver [7]. An aorto-caval lymphadenectomy was done “en bloc” with the mobilization of the head of the pancreas. The origin of the SMA from the aorta was identified and was tumor-free (Fig. 3). An anterograde artery-first approach was done [7, 8] and an accessory right hepatic artery (RHA) originating from SMA was detected and preserved (Fig. 4). Two centimeters distal of the origin of the RHA no clear cleavage plane between the tumor and SMA was found for a distance over 2.5 cm with a free margin of the artery distally at the root of the mesentery (Fig. 5). At this point, the patient was considered resectable. Considering the extension of the disease in the entire gland (mixed-type IPMN) [9], as well as trying to prevent pancreatic leakage in a patient with arterial resection and reconstruction, a decision to do a total pancreatectomy was made. After skeletonization of the hepato-duodenal ligament, transection of the jejunum and duodenum 2 cm distally to the pylorus, a retrograde distal pancreatico-splenectomy was done and the head of the pancreas dissected from the superior mesenteric/portal vein in a complete tumor-free plane. The specimen now remained attached only by the 2.5 cm long contact with the SMA at the level of the uncinate process (Fig. 6). Sterile ice in a surgical towel was placed in the lower sub-mesocolic abdomen in order to reduce the warm ischemia effect and after intra-venous injection of 5000 IU of heparin, the SMA vas clamped proximally and distally to the tumor. The SMA was resected 1 cm proximally and distally to the tumor contact, respectively (for an overall length of about 4 cm), and an end-to-end anastomosis was done using interrupted suture with 5/0 Prolene® (Fig. 7). The overall clamping time of the SMA was 12 min. After reperfusion, the surgical towel with sterile ice was removed and the bowel was inspected to be normal, without signs of edema. In this patient, an extended lymphadenectomy was also performed for technical reasons (i.e., preparation of the SMA for resection and reconstruction) and for staging reasons after the neo-adjuvant treatment (i.e., for assessment of aorto-caval lymph node status). A trans-mesocolic hepatico-jejunostomy and an ante-colic duodeno-jejunostomy were performed and two passive drains were placed. The patient received extended antibiotic and anti-mycotic post-operative prophylaxis and 5000 IU of low-molecular-weight heparin twice a day for 2 weeks. Low dose aspirin was introduced on day 5 after surgery. A CT-scan post-operatively showed normal canalization of the SMA (Fig. 8). The histology confirmed IPMN cancer (pT3N1) with a post-chemotherapy regression grade 3 (10) and without invasion of the arterial wall. The post-operative course was uneventful and the patient was discharged on day 8. Interestingly, no post-operative diarrhea occurred.

## Discussion

The debate on the safety and the oncologic value of arterial resection during pancreatectomy for pancreatic cancer is still open. Data from literature is not consistent and generally comes from small series. The short- and long-term outcomes of pancreatectomies associated with arterial resections have not been encouraging [3]. On the other hand, new more efficient treatments associated with better outcome are now available for patients with pancreatic cancer [5]. However, no data is available regarding the long-term outcome of extended resections in the context of these new and more efficient neo-adjuvant regimens. Preliminary reports, together with some evidence suggesting that surgery may have a survival benefit compared to palliation for patients with locally advanced pancreatic cancer, motivated the scientific international community to be more open to extended resection after adequate medical treatment [2]. New data also shows that the histological regression is not necessarily reflected by radiological CT findings as fibrosis can hardly be differentiated from vital tumor on a CT scan [10, 11]. Furthermore, histological arterial invasion has been confirmed in a median of 26 % of the resected arteries, which illustrates the difficulties of the preoperative assessment of arterial encasement [3]. For this reason, and in the case of a radiological tumor regression after neo-adjuvant treatment, a locally advanced pancreatic cancer should be resected according to the pretreatment CT-demonstrated extent of the tumor, including the vessels involved at that time.

For the above reasons, one may predict that vascular resections during pancreatic surgery will become more frequent in the future, and thus, technically safe and “easy” techniques are necessary. The Cattell-Braasch maneuver has already been demonstrated to be safe and useful for the resection of the superior mesenteric vein (SMV) [7]. The total mobilization of the bowel together with the duodenum and the head of the pancreas not only offer a very good exposure and control of the origin of the SMA but also make the bowel mobile. Hence, the maneuver facilitates a tension-free vascular anastomosis and almost always omits the necessity for placement of an interposition graft both for the SMV and SMA, irrespective of the length of the resected vascular segment. That reduces the vessel clamping time with less ischemia and congestion of the bowel, but also avoids the possible complications related to the graft usage (thrombosis, kinking). This case shows that the Cattell-Braasch maneuver can safely be used for the resection of the SMA with a short clamping time. In the present case, we added local hypothermia, a concept used in transplant surgery. Theoretically, this could reduce the metabolism of the bowel during clamping and the damage caused by ischemia-reperfusion (e.g., edema of the bowel), which represents an increased risk for anastomotic failure. This could be even more beneficial in case of complex simultaneous SMV and SMA resections. Even if this was a single case with short clamping time and no definite conclusions can be made regarding the significance of the local hypothermia for the positive outcome of the procedure, we believe that this technique merits to be reported, since to our knowledge, this method has never been described before in the context of resection of the SMA during pancreatectomy.

## References

[1] Boggi U, Del Chiaro M, Croce C, Vistoli F, Signori S, Moretto C (2009). Prognostic implications of tumor invasion or adhesion to peripancreatic vessels in resected pancreatic cancer. Surgery.

[2] Hartwig W, Vollmer CM, Fingerhut A, Yeo CJ, Neoptolemos JP, Adham M (2014). Extended pancreatectomy in pancreatic ductal adenocarcinoma: definition and consensus of the International Study Group for Pancreatic Surgery (ISGPS). Surgery.

[3] Mollberg N, Rahbari NN, Koch M, Hartwig W, Hoeger Y, Buchler MW (2011). Arterial resection during pancreatectomy for pancreatic cancer: a systematic review and meta-analysis. Ann Surg.

[4] Sgroi MD, Narayan RR, Lane JS, Demirjian A, Kabutey NK, Fujitani RM (2015). Vascular reconstruction plays an important role in the treatment of pancreatic adenocarcinoma. J Vasc Surg.

[5] Khushman M, Dempsey N, Maldonado JC, Loaiza-Bonilla A, Velez M, Carcas L (2015). Full dose neoadjuvant FOLFIRINOX is associated with prolonged survival in patients with locally advanced pancreatic adenocarcinoma. Pancreatology : official journal of the International Association of Pancreatology (IAP) [et al].

[6] Oltean M (2015). Intestinal preservation for transplantation: current status and alternatives for the future. Current opinion in organ transplantation.

[7] Del Chiaro M, Segersvard R, Rangelova E, Coppola A, Scandavini CM, Ansorge C (2015). Cattell-Braasch maneuver combined with artery-first approach for superior mesenteric-portal vein resection during pancreatectomy. Journal of gastrointestinal surgery : official journal of the Society for Surgery of the Alimentary Tract.

[8] Weitz J, Rahbari N, Koch M, Buchler MW (2010). The “artery first” approach for resection of pancreatic head cancer. J Am Coll Surg.

[9] Del Chiaro M, Verbeke C, Salvia R, Kloppel G, Werner J, McKay C (2013). European experts consensus statement on cystic tumours of the pancreas. Digestive and liver disease : official journal of the Italian Society of Gastroenterology and the Italian Association for the Study of the Liver.

[10] Verbeke C, Lohr M, Karlsson JS, Del Chiaro M (2015). Pathology reporting of pancreatic cancer following neoadjuvant therapy: challenges and uncertainties. Cancer Treat Rev.

[11] Ferrone CR, Marchegiani G, Hong TS, Ryan DP, Deshpande V, McDonnell EI (2015). Radiological and surgical implications of neoadjuvant treatment with FOLFIRINOX for locally advanced and borderline resectable pancreatic cancer. Ann Surg.

